# System 2 Diagnostic Process for the Next Generation of Physicians: “Inside” and “Outside” Brain—The Interplay between Human and Machine

**DOI:** 10.3390/diagnostics12020356

**Published:** 2022-01-30

**Authors:** Taro Shimizu

**Affiliations:** Department of Diagnostic and Generalist Medicine, Dokkyo Medical University Hospital, Tochigi 321-0293, Japan; shimizutaro7@gmail.com

**Keywords:** diagnostic strategy, dual-process theory, clinical decision-support system, diagnostic error

## Abstract

Improving diagnosis has been one of the most critical issues in medicine for the last two decades. In the context of the rise of digital health and its augmentation and human diagnostic thinking, it has become necessary to integrate the concept of digital diagnosis into dual-process theory (DPT), which is the fundamental axis of the diagnostic thinking process physicians. Particularly, since the clinical decision support system (CDSS) corresponds to analytical thinking (system 2) in DPT, it is necessary to redefine system 2 to include the CDSS. However, to the best of my knowledge there has been no concrete conceptual model based on this need. The innovation and novelty of this paper are that it redefines system 2 to include new concepts and shows the relationship among the breakdown of system 2. In this definition, system 2 is divided into “inside” and “outside” brains, where “inside” includes symptomatologic, anatomical, biomechanical–physiological, and etiological thinking approaches, and “outside” includes CDSS. Moreover, this paper discusses the actual and possible future interplay between “inside” and “outside.” The author envisions that this paper will serve as a cornerstone for the future development of system 2 diagnostic thinking strategy.

## 1. Redefining System 2

### 1.1. Dissecting System 2—“Inside” and “Outside”

Improving the quality of diagnosis in medical care is considered an essential and urgent issue worldwide [1]. In past decades, research has been conducted on the diagnostic thinking of physicians in the pursuit of high-quality diagnosis. On the other hand, we are now at a stage where the concept of situativity, which encompasses the multiple professions surrounding the physician and patient and the medical environment, will impact the diagnosis [2]. Thus, a multifaceted understanding of diagnosis will be increasingly required for physicians [3].

The basic structure of the modern physicians’ diagnostic thinking has been described as dual-process theory (DPT); the breakdown of DPT is called intuitive system 1 and analytical system 2, with system 2 acting as a counter-check to system 1 to increase the accuracy of diagnosis [4]. In clinical medicine, system 1 diagnosis was often referred to as “non-analytical” diagnosis in the past [5]. This expression suggests that non-analytical (intrinsic) existence is a concept of thought that presupposes an analytical process, system 2. The advantage of system 2 is that it can formulate differential diagnoses more exhaustively and logically than the intuitive system 1. Hence it can operate the diagnostic process safely and comprehensively with fewer omissions of differential diagnoses. If only system 1 is utilized, the diagnostic accuracy may become questionable because system 1 is intuitive and susceptible to various cognitive biases [6]. The disadvantage of system 2 is that it is costly in terms of time and effort (“inside” brain) or cumbersome in terms of requiring external input (“outside” brain) due to the creation of an exhaustive differential list. In addition, it can be less accurate, especially for cases with a common and typical clinical presentation [7].

Surprisingly, as far as the author is aware, the elements of system 2 have not been systematized and categorized so far, despite being known for so long. In addition, there have been no papers exploring the classification of system 2. Besides the digital health field, an element of system 2 has been on the rise [8]. Hence, now is the time for system 2 to be redefined. This paper will articulate a new definition of system 2 classification in diagnostic medicine for physicians in clinical practice and discuss each development and its prospects.

The system 2 diagnostic process, which physicians directly use in the field, can be divided into two major categories: “inside” brain and “outside” brain system 2 processes. (Table 1).

### 1.2. Symptomatologic Approach

The “inside” brain process can be further divided into four parts: symptomatologic approach, anatomical approach, biomechanical–physiological approach, and etiological approach.

The symptomatologic approach organizes diagnostic possibilities using analytical techniques specific to each symptom (e.g., syncope, dyspnea, headache, back pain, bloody stool, etc.) or phenomenon. For example, syncope is a transient loss of consciousness caused by transient global cerebral hypoperfusion [9]. The causes of syncope can be analytically stratified into all diseases (mainly cardiogenic) that cause shock, widespread lesions that can cause whole-brain ischemia (bilateral internal carotid artery dissection, subarachnoid hemorrhage), blood flow disturbances affecting the ascending reticular activating system, including the thalamus (e.g., cerebrovascular diseases involving posterior circulation or perforating branch), and syncope-like symptoms (hypoglycemia, hypoxia, psychogenic pseudo-syncope, etc.) [10]. Clinical phenomena (e.g., a recurrent event) can also be categorized in their unique ways. In this way, this approach is a precise analysis that systematically considers each symptom or phenomenon. In the author’s opinion, this approach is the most frequently used analytical approach in system 2 for common syndromes.

### 1.3. Anatomical Approach

The anatomical approach is an effective way to identify the location of the anatomical structure or organ causing the problem while “imagining” the cross-section of the CT scan in physicians’ minds [11,12]. For example, a physician might combine a physical examination with this approach to detect that a headache is caused by inflammation of the temporal artery or that abdominal pain is caused by entrapment of the anterior cutaneous nerve in the abdominal wall or by an enlarged abdominal aorta. The anatomical approach is also beneficial in medical education because it promotes the concept of a hypothesis-driven physical examination, where the patient imagines (reasoning) the anatomical lesion and then actually goes to look for the finding in the physical examination [13]. The anatomical approach also covers microscopic approaches to determine which anatomical structures are affected (e.g., determining which subcutaneous structures are affected when examining palpable purpura or livedo racemosa). The anatomical approach is efficient when symptoms are localized.

### 1.4. Biomechanical-Physiological Approach

The biomechanical approach considers the dynamic relationships of biological structures that are not covered by the anatomical approach. The physiological approach deals with the functions of the human body, focusing on biochemical and electrophysiological functions. The biomechanical and physiological approaches are grouped because they sometimes share the same medical problem, even if this is not always the case. For example, if a physician sees chronic numbness induced by limb movement, the physician considers nerve root or spinal cord damage due to dynamic effects of the spine. Then the physician considers the type of peripheral nerve fibers affected based on the quality of numbness; both approaches become necessary simultaneously.

### 1.5. Etiological Approach

The etiological approach is a method that examines the differential diagnosis for each functional organ system. Based on the patient’s information, the physician reviews the differential diagnosis by each category, such as neoplastic, vascular, and infectious diseases. For example, a 76-year-old man at vascular risk with rapidly progressive loss of consciousness may have a subdural hematoma if vascular, metastasis of a malignant tumor if neoplastic, or herpes encephalitis if infectious. The author uses the mnemonic code MEDICINE to organize and classify the categories in etiological diagnosis (Table 2).

This approach is versatile and can be used in any diagnostic situation. However, this approach may be the most time-consuming because of the thorough consideration of all possibilities.

### 1.6. “ Outside” Brain

Apart from the “inside” brain approach, the “outside” brain approach refers to the digital approach centered on CDSS (Clinical decision-support system). In the digital approach, diagnostic AI software that incorporates deep learning and search engines such as Pubmed and Google are used to help bring to light possible diagnoses that physicians cannot recall. When the CDSS derives a diagnosis through the input of medical history and physical findings, it has been a challenge for AI to reason why the diagnosis was derived. Still, the development of explainable AI is underway to explain the “thinking” process of the CDSS [14]. Consequently, for the “inside” brain, interplay (or more precisely, mutual supervision) with “outside” brains is becoming more available.

Moreover, the digital system, which is the “outside” brain, may help train the physician’s “inside” diagnostic thinking, as it can serve as an objective guide for human judgment from the outside. Furthermore, suppose the “inside” strategic model is applied to the “outside” through machine learning. In that case, it is expected to increase the accuracy of diagnosis through the collaboration between “inside” and “outside”. A pivot and cluster strategy (PCS) is an “inside” diagnostic thinking model that combines systems 1 and 2 to prevent diagnostic omissions by developing a differential diagnosis of clinical phenotypes similar to the first diagnosis that intuitively comes to mind [15,16]. This PCS has been applied to machine learning to develop an “outside” CDSS model [17]. Such an interplay example would be applied in another way, such as a horizontal and vertical tracing strategy (HVT: another cognitive forcing diagnostic strategy based on a particular diagnosis or syndrome that provides a comprehensive picture of the underlying pathology and comorbidities [18,19]) would be an excellent candidate to be applied to CDSS, because HVT resembles PCS in light of cognitive forcing strategy. Hence HVT can be incorporated into CDSS without technological challenge.

### 1.7. An Overview of the Proposed System 2

**Figure 1:**
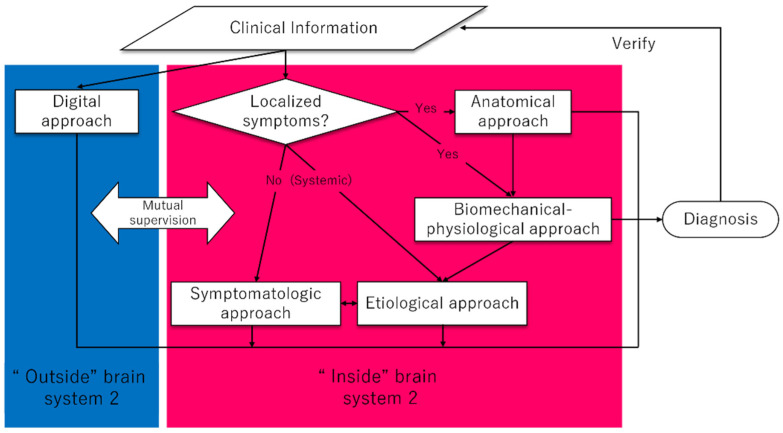
A proposed algorithm of system 2 approaches.

An overview of the involvement of the variable system 2 approaches introduced so far is shown in Figure 1.

Figure 1 shows a proposal of a flowchart when using system 2. The blue area in the figure is the area of the “outside” brain, and the red area in the figure is the area of the “inside” brain. Based on the clinical information obtained from the patient, system 2 of the physician’s thinking is initiated (“inside” brain). As the most effective thinking, if the symptoms are confined to localized areas of the body, it is helpful to utilize the anatomical approach. This is because localized symptoms can be identified by anatomical analysis. When the causative organ is identified, it can lead directly to the diagnosis, and when the symptom involves a biomechanical–physiological aspect, the biomechanical–physiological aspect approach follows after the anatomical approach. When the symptom mainly has a biomechanical–physiological characteristic, physicians should directly think of the biomechanical–physiological aspect related to the focal site of the body. After these approaches, if the affected anatomical area or organs need etiological consideration, such as inflammation, tumor, or obstruction, the final diagnosis can be identified with the etiological approach (e.g., shoulder pain with passive movement prompts localization of intraarticular pathology, and the possible etiologies include infection, bleeding, or autoimmunity; fainting with specific upper arm movements can localize the subclavian artery as a suspected organ (subclavian artery steal syndrome), and the possible etiologies include atherosclerosis, inflammation, or trauma). When the symptom is systemic rather than localized, the diagnostic analysis will proceed to etiologic or symptomatologic approaches. At the same time as utilizing this “inside” brain, physicians could utilize a digital approach (“outside” brain) to monitor the “inside” brain or provide optimal solutions for diagnoses that were inconclusive in the “inside” brain. It will be possible to use the digital approach (“outside” brain) to monitor the “inside” brain or to provide the optimal solution for reaching a diagnosis that is inconclusive only with the “inside” brain approaches.

This flowchart illustrates the typical use of system 2 from the front-line physicians’ perspectives, but in practice, other flows might be added to this flowchart. Besides, once identified the diagnosis must be validated for accuracy by comparing the diagnosis to the clinical information already obtained so far. This flow is also noted in the above flowchart. By making it a habit to consciously use these five system 2 approaches, either alone or in combination, solid diagnostic accuracy should be expected even in diagnostically challenging cases.

## 2. How to Nurture the Effective Application of system 2

### 2.1. Building Medical Knowledge as a Basis of System 2 Diagnostic Thinking

Medical knowledge is the source of a physician’s intellectual fitness [8]. Increasing and structuring medical knowledge has been shown to produce consistent positive results in improving diagnosis [20], which is also an essential factor in the effective operation of system 2. For example, the approaches that comprise system 2 include the anatomical approach and the biomechanical–physiological approach. The more detailed the basic medical knowledge, such as anatomy, physiology, biochemistry, and pharmacology, the more precise the analysis becomes. As a result, more accurate system 2 thinking can be achieved, strengthening the foundation of diagnostic capability. Besides, explaining a patient’s symptoms against the background of such basic medical knowledge is vital in acquiring diagnostic reasoning [21]. It may be a painstaking task for the front-line physicians to open and review textbooks of basic medicine. However, as a large part of system 2 depends on knowledge of basic medicine, it must be said that regular review of basic medicine is essential for the lifelong education of physicians.

It is obvious that clinical knowledge is necessary because it is directly related to diagnosis; among the items that make up system 2, it is the etiological approach and the symptomatologic approach that particularly entail clinical medicine knowledge. Clinical knowledge is usually organized by organ system or disease etiology, and the construction of etiological approaches may not cause many difficulties. On the other hand, if the information is systematically organized by symptom, physicians must organize it without a corresponding review article. What is essential here is a detailed knowledge of each disease, or more precisely, an accurate understanding of the illness script of each disease. This is because if the illness script is accurately understood, the task of reclassifying the disease by etiology or by symptomatology will be more accurate. Another reason for the importance of accurately capturing the illness script for each disease is that this understanding defines the accuracy of the diagnosis at the final stage of narrowing down the list of potential differential diseases. For example, if two diseases are similar clinical phenotypes, they will be considered differential diagnoses, and differentiation will be necessary. Recognizing what findings are common to the two diseases and what are different will affect making a precise differential diagnosis [15,16]. Furthermore, having a solid typological disease illness script, especially for diseases the physician has little or no experience with, will help rapidly calibrate the diagnostic process by metacognition when an atypical case is encountered. The author would argue that mastery of typical disease illness scripts of diseases is based on two plans: a daily review of textbooks regarding a disease encountered at the bedside, and study of case reports on clinical variants and atypical symptoms of a specific disease encountered.

Therefore, the author would propose it is essential that building medical knowledge is based on carefully reviewing cases encountered daily, continuing to learn basic medicine from textbooks and atlases and clinical medicine from textbooks, review articles and case reports as reference materials.

### 2.2. The Importance of Calibration, Reflective Practice, and Adaptive Training

As shown in the flowchart, a diagnosis should always be treated as provisional unless it is definitive and should be calibrated to the clinical situation on a case-by-case basis. For example, suppose the diagnosis turns out to be an error. In that case, it is necessary to reflect and calibrate the physician’s behavior and examine the physician’s cognitive frame of mind that led to the behavior [22]. Diagnostic calibration means that the diagnostician’s confidence level in the individual diagnosis is adjusted to match the actual diagnosis [23]. This feedback process should be given regularly.

Reflective practice is advocated to work in improving diagnosis effectively [24]. The reflection intervention in the calibration action has been shown to positively impact diagnostic accuracy [25,26]. Reflection has been effective in complicated cases [27].

Reflection has three categories: Reflection in action, Reflection on action, and Reflection for action. Applying all of them should be essential to achieve expertise for the exponential growth of diagnostic expertise [28].

Besides, incorporating the Safety 2 concept would be encouraged in performing Reflection [29]. To cope with complex situations, it is necessary to be proactive, always anticipating changes and new events. Therefore, as well as identifying what went wrong and the factors that contributed to it (Safety 1), it is crucial to envision the “complements” to the error cases that are usually considered, such as understanding how things usually work, thereby leading to a balanced reflection. In particular, considering why things went well and how resilient adjustments were made to cases that have gone well can help us reflect positively.

In the case of performing Reflection to colleagues, it is helpful to apply “debriefing with good judgment”, which is helpful in reflective practice when reflecting with peers or advanced students [30]. This teaching method focuses on understanding the thinking frame of the learner’s thinking without compromising learners’ motivation and on making the instructor’s ideas explicit. Debriefing with good judgment is essential because it reduces psychological stress for both the teacher and the learner, strengthening metacognition.

In addition to Reflection, adaptive training that incorporates lateral thinking and mindfulness is said to help improve metacognition to address factors that influence complex clinical reasoning [31,32].

### 2.3. Maximizing Collective Intelligence

The above flowchart shows system 2 in an individual’s brain, but to make it even more effective, it would be better to run system 2 with multiple brains. The usefulness of collective intelligence (CI) has long been noted [33]. CI is when a group of people brings together the members’ knowledge and skills to solve a problem requiring complex cognitive processes. The performance of CI has been shown to outperform individual performance in the diagnostic work of physicians [34]. Particularly, similarity in diagnostic accuracy proved pivotal for CI, given that there is no significant difference in diagnostic accuracy between physicians. In other words, it would be possible to provide a stable and high-quality diagnosis by pooling together a group of physicians with relatively homogeneous diagnostic skills. CI is also a helpful strategy from a metacognition perspective. In fact, given that due to “noise”, the decision-making randomness of each individual [35], the diagnostic performance by the best individual diagnostician can be more volatile than that of the average of the group composed of individuals, it is safer to spread the risk by diluting the noise with collective intelligence. As an extension of the concept of CI, it would be helpful to add intelligence other than the diagnosis of a group of experts based on collective intelligence. Opinions of physicians and other health professionals who have previously examined the patient and those of patients and surrounding personnel who are non-experts can be reflected in subsequent reflections, leading to a better calibration of the diagnosis [2,36].

Furthermore, healthcare organizations of various sizes would need to support the continuation and development of team-based endeavors involving CI. For instance, within a single institution or a network of institutions, time, space, money, and personnel can be invested to formally support such a diverse team’s diagnostic activities [1]. Such a perspective is in keeping with the context of “situativity”, in which the diagnosis is shaped by the complex and dynamic interaction between people and their environment.

### 2.4. The Key for Exceeding the Confronting Health Quality Problem from a System 2 Viewpoint

Cost, speed, and accuracy are essential factors in diagnosis, but the effectiveness of these factors in system 2 has not been well established. Especially for CDSS, which is the most promising system 2, while there have been single-center reports that CDSS has contributed to cost reduction [37], accurate diagnosis [38], reduced diagnostic costs, shortened diagnostic time, and fewer unnecessary invasive procedures [39]. The results of systematic reviews report that there has still been insufficient evidence of consequences on clinical, economic, workload, and efficiency [40]. Similarly, consistent results of the CDSS for individualized institutional groups and diseases have not been shown [41]. As mentioned in these papers, the lack of established effectiveness is due to the scarcity of accumulated evidence. To overcome these situations, further accumulation of evidence is required. The author would like to emphasize here that in addition to the accumulation of evidence from multicenter prospective studies, it is necessary to accumulate evidence by disease and institutional setting (e.g., hospital, clinic, emergency room, intensive care unit, etc.). In order to make a large-scale study possible, each facility would first need to implement the CDSS, even on a trial basis. Since AI–human collaboration and augmentation is the foreseeable future, it is consistent with the demands that each facility considers implementing CDSS. It is also necessary to promote the digital transformation in each medical institution for the coming singularity. Besides, the improvement of CDSS is expected to have a positive impact on each medical professional’s classic system 2, the “inside” brain system 2, as a result of interaction between “inside” and “outside”. CDSS is an element that is part of system 2, an improvement in CDSS will further strengthen system 2 thinking and positively affect overall diagnostic performance.

Other than the evidence issue, opposition and distrust of CDSS alerts remain factors that could hinder the development of CDSS [42]. However, as the effectiveness of CDSS, which will be verified in the future, becomes more widely recognized and trusted, the distrust is likely to dissipate.

Considering the barriers that arise in the physician–patient relationship is essential in improving health quality [2]. Even in system 2, there are barriers from the perspective of physicians and patients. A typical example is the “Outside” brain system 2, such as CDSS, where the patient may not be satisfied with the results because they are not output information from a human physician. However, it is expected that the psychological barriers to CDSS can be removed by explaining to patients that the current CDSS serves to assist and monitor human decision-making and does not make decisions on its own.

### 2.5. A Next-Generation Concept That May Compensate for the Weakness of DPT

DPT is a thinking strategy that breaks through the stereotype that diagnosis is based solely on experience and tacit knowledge. With the proposal of DPT, the impression that diagnosis is a particular skill that a limited number of specialists can only achieve has been dispelled. Additionally, system 2 has provided inexperienced physicians with the technology to obtain a reproducible and accurate diagnosis.

The idea that the DPT model can tentatively explain how we think about diagnosis is a product of new insights in medicine. However, it is not clear whether DPT will remain the optimal universal diagnostic model in the future. If we are uncomfortable with the idea that the dualism of DPT provides a complete explanation for diagnostic decision-making, we need to apply or create a new concept. For example, neuroscience’s predictive process (PP) has recently attracted much attention. PP is the concept from a neural network, which learns to make the residuals between the error signal output by processing the lower layers’ input and predicting that error signal to zero. This concept is explained by a unifying model of thinking processes, including metacognition, which, if applied, could be used as a more natural process than DPT [43]. The concept of PP is highly affinitive to the digital approach, including machine learning. The Digital approach is currently classified as system 2 given DPT. When a thinking model such as PP, which breaks the framework of DPT, emerges and is accepted and implemented by society, it may cause a fundamental change in the way we think about diagnosis.

There is still room for the “inside” brain to develop. Meanwhile, the “outside” brain is expected to develop independently of the “outside” brain as the CDSS develops further. Simultaneously, due to the interplay between the two, it is expected that the development of the “outside” brain will have a positive impact on the “inside” brain and that the synergy between the “outside” and “inside” brains will lead to the development of system 2.

## 3. Conclusions

The innovation and novelty of this paper are as follows: first, it redefines system 2 with a new concept and provides a breakdown of the elements that make up system 2, which had not been clearly defined before. Second, the paper presents a flowchart of the priorities and relationships of each element of the “inside” brain. Third, it discusses the interaction between “inside” and “outside” and its future possibilities. Fourth, this paper presents the most comprehensive system 2 approach, the etiological approach, as a practical mnemonic and a practical form of “MEDICINE.” Fifth, this paper introduces the current system 2 approach’s problems regarding health quality and suggests ways to improve them. The author hopes this paper will become a cornerstone for developing system 2 diagnostic thinking strategies. Additionally, based on this paper, future experimental evidence evaluating the advantage of applying the concept and flowchart should be warranted.

## Figures and Tables

**Table 1:** Overview of system 2 approaches.

**“Inside” Brain**
Symptomatologic approach
Anatomical approach
Biomechanical-physiological approach
Etiological approach
**“Outside” Brain**
Digital approach

**Table 2:** The MEDICINE mnemonic code for etiological diagnostic approach.

**M**	ental
**E**	ndocrine/Metabolic: **GLUT-HUBS** *
**D**	rug (Toxin, Drug, Nutritional, Lytes)
**I**	nflammation: **I**nfection and **I**mmune
**C**	urrent disturbance: **ABCDEF-RUV** †
**I**	atrogenic (including Foreign body)/Traumatic
**N**	eoplastic-Infiltrative
**E**	lse: **E**pidemiologic, **E**ssential, **E**ctopic, **E**nvironmental

*: **G**lucose, **L**iver, **U**remia, **T**hyroid, **H**PA-Axis, **U**ric acid metabolism, **B**one metabolism, **S**carce diseases; †: **A**irway, **B**ile duct, **C**SF, **D**igestive, **E**lectric (Neuro), **F**istula, **R**eproductive, **U**rinary tract, **V**ascular.
